# Hyaluronic Acid–Stabilized Fe_3_O_4_ Nanoparticles for Promoting *In Vivo* Magnetic Resonance Imaging of Tumors

**DOI:** 10.3389/fphar.2022.918819

**Published:** 2022-07-15

**Authors:** Weijie Zhang, Zhongyue Zhang, Shitong Lou, Zhiwei Chang, Baohong Wen, Tao Zhang

**Affiliations:** ^1^ Department of Oncology, The First Affiliated Hospital of Zhengzhou University, Zhengzhou, China; ^2^ Department of MRI, The First Affiliated Hospital of Zhengzhou University, Zhengzhou, China; ^3^ College of Pharmacy, Xinxiang Medical University, Xinxiang, China

**Keywords:** iron oxide nanoparticles, magnetic resonance imaging, hyaluronic acid, HeLa cell/tumor, active targeting

## Abstract

The use of iron oxide (Fe_3_O_4_) nanoparticles as novel contrast agents for magnetic resonance imaging (MRI) has attracted great interest due to their high *r*_2_ relaxivity. However, both poor colloidal stability and lack of effective targeting ability have impeded their further expansion in the clinics. Here, we reported the creation of hyaluronic acid (HA)-stabilized Fe_3_O_4_ nanoparticles prepared by a hydrothermal co-precipitation method and followed by electrostatic adsorption of HA onto the nanoparticle surface. The water-soluble HA functions not only as a stabilizer but also as a targeting ligand with high affinity for the CD44 receptor overexpressed in many tumors. The resulting HA-stabilized Fe_3_O_4_ nanoparticles have an estimated size of sub-20 nm as observed by transmission electron microscopy (TEM) imaging and exhibited long-term colloidal stability in aqueous solution. We found that the nanoparticles are hemocompatible and cytocompatible under certain concentrations. As verified by quantifying the cellular uptake, the Fe_3_O_4_@HA nanoparticles were able to target a model cell line (HeLa cells) overexpressing the CD44 receptor through an active pathway. In addition, we showed that the nanoparticles can be used as effective contrast agents for MRI both *in vitro* in HeLa cells and *in vivo* in a xenografted HeLa tumor model in rodents. We believe that our findings shed important light on the use of active targeting ligands to improve the contrast of lesion for tumor-specific MRI in the nano-based diagnosis systems.

## Introduction

Early and precise diagnosis is essential for treatment of cancer in the clinic, which continuously pushes the need for advanced imaging modalities and contrast agents. Magnetic resonance imaging (MRI) has been considered as one of the most effective and valuable *in vivo* bioimaging techniques because of its noninvasive and high-resolution features (Harisinghani et al., 2003; Sim et al., 2020). Traditional contrast agents for MRI are often small molecules with chelated metals (Caravan et al., 1999; Lohrke et al., 2016; Wahsner et al., 2019), such as Gd(III) or Mn(II). However, these molecular imaging agents suffer from rapid clearance and low efficiency due to their small-molecule nature (Lee et al., 2010). The emergence of nanotechnology has simulated the use of nanoparticles as novel contrast agents and brought additional benefits due to the enhanced permeability and retention effect (EPR) (Hifumi et al., 2006; Bridot et al., 2007; Brigger et al., 2012; Oliveira et al., 2014; Swierczewska et al., 2016; Wei et al., 2017; Farzin et al., 2020; Leon-Janampa et al., 2020; Mitchell et al., 2021). Among them, iron oxide (Fe_3_O_4_) nanoparticles are the classic ones used in both *T*_1_-weighted and *T*_2_-weighted MRI (Ma et al., 2017; Shen et al., 2017). The ultrasmall ones with diameters smaller than 5 nm are used as positive contrast agents for *T*_1_-weighted MRI, while the bigger ones larger than 10 nm are potentially used as negative contrast agents for *T*_2_-weighted MRI due to their high *r*_2_ relaxivity (Hu et al., 2011; Lee and Hyeon, 2012; Lee et al., 2015; Martinkova et al., 2018; Dadfar et al., 2019; Souri et al., 2022). Nevertheless, one major hurdle is that Fe_3_O_4_ nanoparticles easily aggregate and precipitate in the solution (Yu et al., 2006; Kim et al., 2011); the other one is that the use of bare Fe_3_O_4_ nanoparticles only benefits from passive targeting as a result of the EPR effect in leaky vasculature and poor lymphatic drainage (Maeda, 2010; Barreto et al., 2011; Luo et al., 2015). This approach does not apply for tumor-specific MRI. Therefore, to increase the efficiency and specificity, it is crucial to create stable nanoparticles with active tumor targeting properties.

Anchoring water-soluble polymers, for example, polyethylene glycol (PEG) (Yallapu et al., 2010; Hu et al., 2011; Li Z. et al., 2013; Luo et al., 2015), polyethyleneimine (PEI) (Mcbain et al., 2007; Liu et al., 2011; Li J. et al., 2013), chitosan (Zhi et al., 2006; Kievit et al., 2009; Shi et al., 2012; Khmara et al., 2019), and dextran (Tassa et al., 2011; Easo and Mohanan, 2013; Naha et al., 2019; Zhao et al., 2021), onto the surface of Fe_3_O_4_ nanoparticles is a proven strategy to stabilize the nanoparticles and avoid the formation of large aggregates on precipitation. Although this strategy could help to some extent, the functionalized Fe_3_O_4_ nanoparticles still lack the targeting ability. Hyaluronic acid (HA) is a water-soluble glycosaminoglycan with repeating units of d-glucuronic acid and N-acetyl-d-glucosamine (Lapcik et al., 1998; Choi et al., 2009; Della Sala et al., 2022). It is a natural polymer involved in many important physiological processes, such as wound healing, tissue regeneration, and joint lubrication. It has also been identified as a targeting auxiliary with high affinity for the CD44 receptor (Eliaz and Szoka, 2001; Toole, 2004; Ju et al., 2016; Lee et al., 2016), which is overexpressed in a variety of tumors. Therefore, HA could be used as a targeting ligand for both enhanced imaging and therapy (Lee et al., 2008a; Lee et al., 2008b; Li et al., 2014; Xiao et al., 2015; Zhang et al., 2020; Zhou et al., 2021).

**FIGURE 1:**
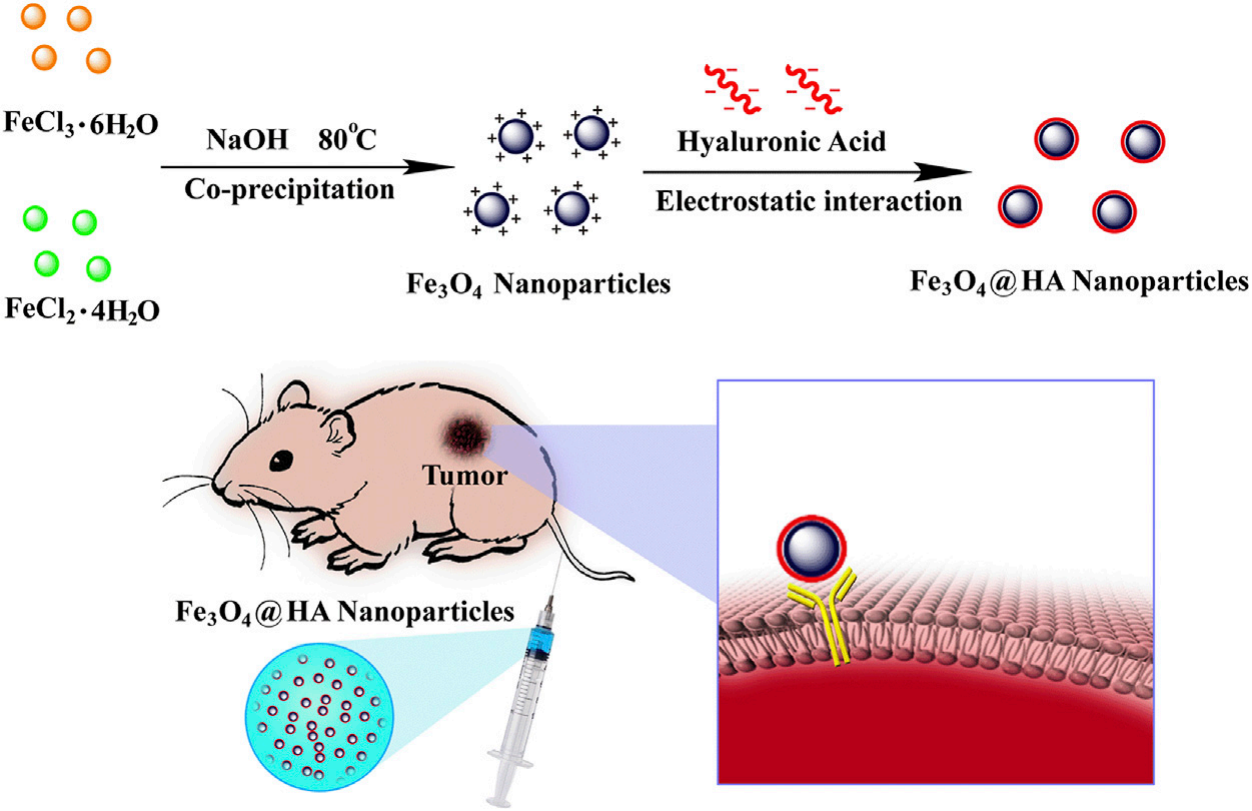
Schematic illustration of synthesis and preparation of HA-stabilized Fe_3_O_4_ nanoparticles and Fe_3_O_4_@HA nanoparticles targeting the tumor *via* active binding with the receptor.

With the aim of improving the contrast of lesions, we reported in this work the creation of HA-stabilized Fe_3_O_4_ nanoparticles and their applications in enhanced MRI of tumors. Fe_3_O_4_ nanoparticles were first synthesized *via* a co-precipitation method (Ding et al., 2016). The resulting nanoparticles possess positively charged surface chemistries, which were subsequently stabilized *via* HA through electrostatic interactions (Figure 1). Some literature reports have shown the modification of HA onto the surface of Fe_3_O_4_ nanoparticles by chemical conjugation (Li et al., 2014; Gong et al., 2019; Luo et al., 2019; Zhang et al., 2020). However, this method requires tedious chemical reactions, which could result in low yield, low functionalization density due to inefficient reaction, and batch-to-batch variations in modification density. Taking advantage of electrostatic interactions potentially contributes to the reproducibility of the system since the functionalization density mostly depends on the surface charge of the Fe_3_O_4_ nanoparticles. The HA-coated nanoparticles (Fe_3_O_4_@HA) were characterized with a variety of techniques, such as X-ray diffraction (XRD), Fourier transform infrared spectrometry (FTIR), thermogravimetric analysis (TGA), transmission electron microscopy (TEM), and dynamic light scattering (DLS), to verify the success in synthesis and stabilization. Through a series of *in vitro* studies, we showed that Fe_3_O_4_@HA nanoparticles are biocompatible and hemocompatible. We demonstrated the targeting ability of Fe_3_O_4_@HA nanoparticles at the cellular level *via* comparing cellular uptake of nanoparticles in HA active and blocked modes, respectively. More importantly, Fe_3_O_4_@HA showed much enhanced contrast for MRI in a cervical tumor model in rodents.

## Results and Discussion

### Synthesis and Characterization of Fe_3_O_4_@HA Nanoparticles

**FIGURE 2:**
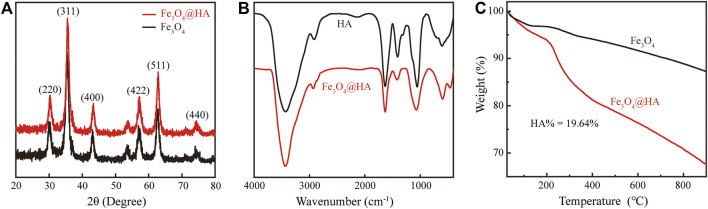
Characterization of Fe_3_O_4_@HA nanoparticles. **(A)** XRD pattern of bare Fe_3_O_4_ and Fe_3_O_4_@HA nanoparticles. **(B)** FTIR curves of HA and Fe_3_O_4_@HA nanoparticles. **(C)** TGA analysis of bare Fe_3_O_4_ and Fe_3_O_4_@HA nanoparticles.

Fe_3_O_4_ nanoparticles were first synthesized *via* a hydrothermal co-precipitation approach (Ding et al., 2016). HA was wrapped onto the surface of nanoparticles through electrostatic interactions (Figure 1). XRD was used to investigate the patterns of nanoparticles. Both bare Fe_3_O_4_ (black) and Fe_3_O_4_@HA (red) nanoparticles showed same peaks in (220), (311), (400), (422), (511), and (440) planes (Figure 2A), indicating that the incorporation of HA into the system happened in a physical absorption way rather than a chemical reaction. FTIR results (Figure 2B) showed the existence of characteristic signals of HA in Fe_3_O_4_@HA nanoparticles, suggesting successful functionalization of HA corona. Furthermore, TGA measurements were used to quantitatively identify the content of HA binding on the surface of Fe_3_O_4_@HA nanoparticles. Compared with bare Fe_3_O_4_ nanoparticles, the modified Fe_3_O_4_@HA nanoparticles exhibited an increase in weight loss from 12.75 to 32.39% (Figure 2C). The content of HA on the surface of nanoparticles is 19.64%. These results together indicate that we have successfully synthesized Fe_3_O_4_@HA nanoparticles with an HA loading of around 20% by weight.

**FIGURE 3:**
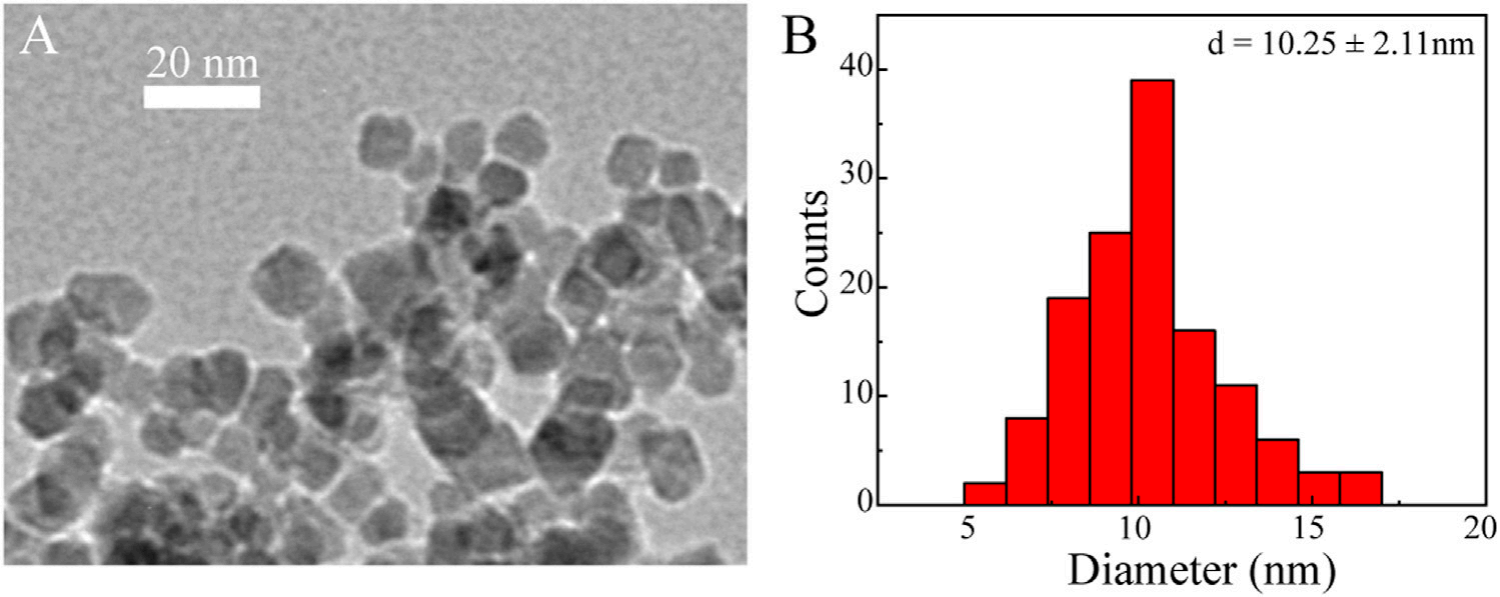
Representative TEM image **(A)** and histogram of size distribution **(B)** of Fe_3_O_4_@HA nanoparticles. Spherical nanoparticles were observed with an estimated size of around 10.25 ± 2.11 nm (*n* = 132). As the particles were not stained, the HA corona was almost invisible. The actual size of Fe_3_O_4_@HA should be considered larger than the size measured here.

**FIGURE 4:**
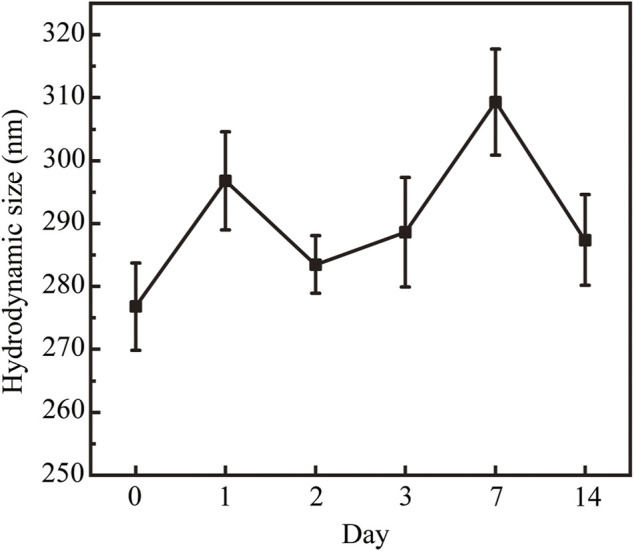
Hydrodynamic size of the Fe_3_O_4_@HA nanoparticles and their physical stability over 14 days. As a result of slight aggregation, the hydrodynamic size is between 270–310 nm. Monitoring the hydrodynamic size over 14 days showed that the system was stable and did not form super large aggregations or precipitate out within 14 days. Data are calculated from three parallel measurements and presented as mean ± SD.

We next characterized the morphology and size of the resulting Fe_3_O_4_@HA nanoparticles. TEM imaging clearly revealed spherical nanoparticles with a uniform size distribution (Figure 3A). The size of the nanoparticles was further measured by ImageJ and estimated to be around 10.25 ± 2.11 nm (Figure 3B). Since we did not stain the nanoparticles, the contrast of the HA corona should be relatively low and even negligible. Therefore, the size we measured here is possibly the size of bare Fe_3_O_4_ itself. The actual size of Fe_3_O_4_@HA should be considered larger than the size here with the extra HA corona. In addition, we examined the hydrodynamic size and physical stability of Fe_3_O_4_@HA in PBS buffer *via* DLS. The results showed that the hydrodynamic size of Fe_3_O_4_@HA nanoparticles is in between 270–310 nm (Figure 4), which is much larger than the size measured by TEM. This is plausibly caused by slight aggregation or interaction among nanoparticles in the solution, as also observed by others in similar systems (Li et al., 2014). The intensity averaged particle size measured by DLS often reflects a bit more on large-sized particles even though the majority are small ones (Fischer and Schmidt, 2016). With TEM imaging, we were able to identify the single nanoparticles. Monitoring the hydrodynamic size of Fe_3_O_4_@HA for 14 days (Figure 4) showed that the system did not form a super large aggregation or precipitate out within this period of time. Therefore, the bare Fe_3_O_4_ nanoparticles were successfully stabilized by coated HA. The zeta potential of Fe_3_O_4_@HA was measured as 32.3 ± 0.8 mV.

### Measurements of *T*_2_ Relaxivity of Fe_3_O_4_@HA Nanoparticles

**FIGURE 5:**
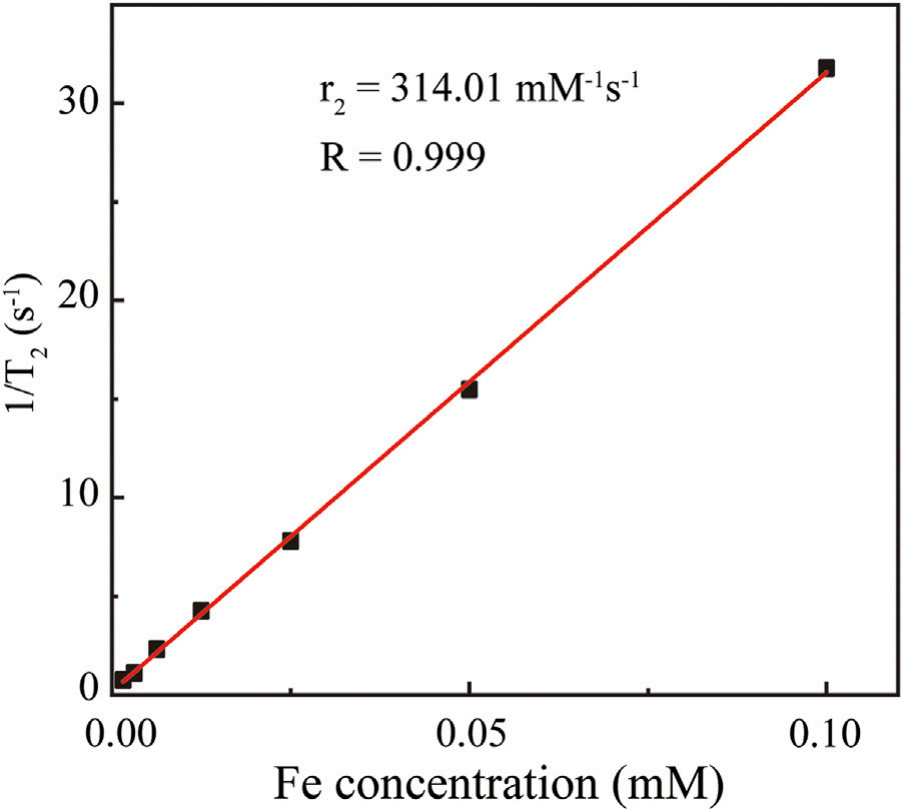
Linear fitting of 1/*T*_2_ *vs*. Fe concentration. The Fe concentrations are 0.00156, 0.00313, 0.00625, 0.0125, 0.025, 0.05, and 0.1 mM. The resulting transverse relaxivity *r*_2_ was estimated as 314.01 mM^−1^s^−1^.

Since magnetic Fe_3_O_4_ nanoparticles can shorten the transverse *T*_2_ relaxation time of water protons so as to enhance the contrast, we next characterized the *T*_2_ of protons in the Fe_3_O_4_@HA solutions of various Fe concentrations. The results (Figure 5) clearly showed that with the increase in the Fe concentration, Fe_3_O_4_@HA nanoparticles were able to cause a decrease in the magnetic resonance (MR) intensity in the *T*_2_-weighted mode. The transverse relaxivity *r*_2_ (the transverse relaxation rate per mM of iron) was further calculated by linear fitting of the relaxation rate 1/*T*_2_ *vs*. Fe concentration (Figure 5). The *r*_2_ of the Fe_3_O_4_@HA nanoparticles was estimated at around 314 mM^−1^s^−1^. This number is higher than the numbers reported in the literature (Shi et al., 2008; Cai et al., 2012; Li J. et al., 2013; Li et al., 2014), which could be possibly caused by the thinner layer of HA corona due to electrostatic interactions compared with the chemical conjugated ones. Water molecules can penetrate easily through the thinner HA corona and interact with Fe_3_O_4_ in the core of the nanoparticles. These results suggested that Fe_3_O_4_@HA nanoparticles with such high *r*_2_ can be used as a potential candidate for *T*_2_-weighted MRI.

### Hemolysis Measurements

**FIGURE 6:**
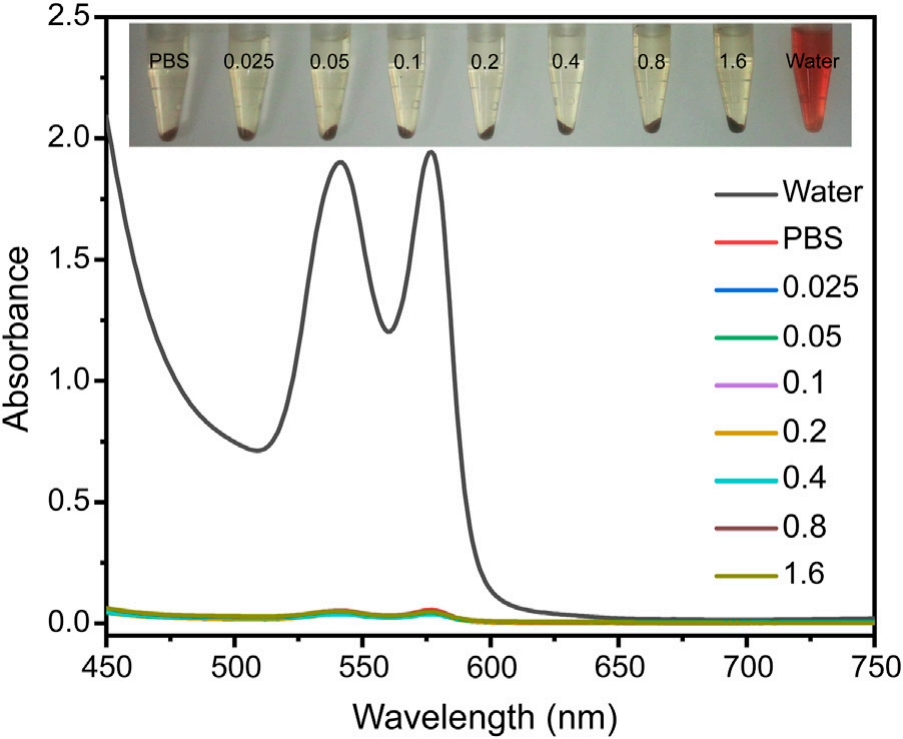
Hemolysis assay of Fe_3_O_4_@HA nanoparticles was measured at Fe concentrations of 0.025, 0.05, 0.1, 0.2, 0.4, 0.8, and 1.6 mM with water and PBS as controls. The insert graph on the top is a photo of RBCs incubated with different solutions for 2 h and subsequently centrifuged. The UV-Vis absorptions of the supernatants were then collected.

Given that all the contrast agents are eventually used for *in vivo* detection and diagnosis, they should be extremely biocompatible with the body. We therefore investigated the hemocompatibility of the Fe_3_O_4_@HA nanoparticles, which is one of the crucial prerequisites before the *in vivo* studies. The hemolysis assay was performed at different Fe concentrations (0.025, 0.05, 0.1, 0.2, 0.4, 0.8, and 1.6 mM) with water and PBS as controls (Figure 6) for 2 h incubation at 37°C. The absorbents were collected after incubating Fe_3_O_4_@HA nanoparticles with RBC suspension (Figure 6). Along with PBS solution, we did not notice the strong hemolysis effect in the samples with Fe_3_O_4_@HA, while the water control showed an obvious hemolysis phenomenon. By comparing the absorbance at 541 nm, we found that the percentage of hemolysis is almost negligible (less than 3%) under the current Fe concentrations. Therefore, we concluded that our Fe_3_O_4_@HA nanoparticles were hemocompatible, warranting further investigation *in vivo*.

### *In Vitro* Cytotoxicity Assay and Cellular Uptake

**FIGURE 7:**
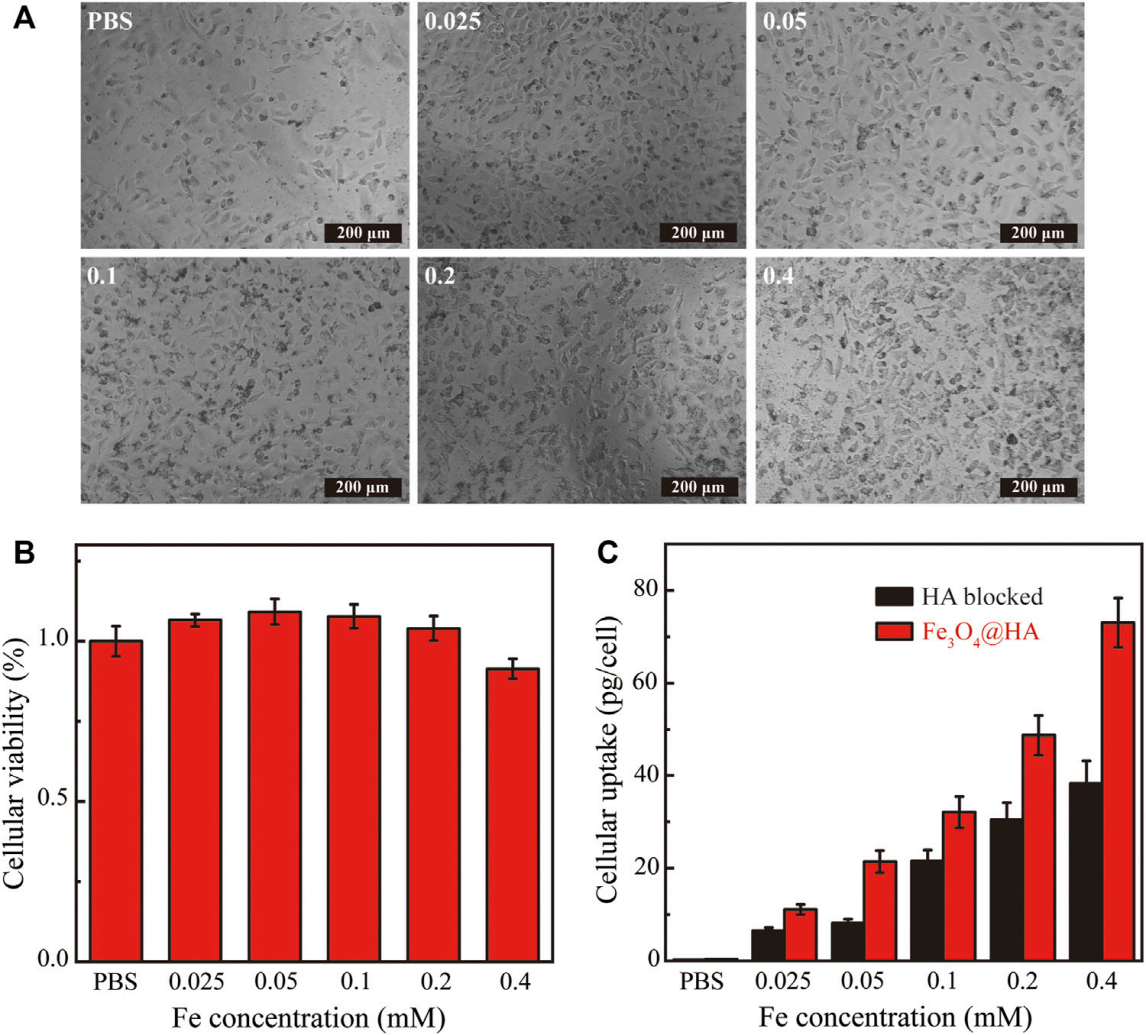
**(A)** Phase contrast microscopic images of HeLa cells incubated with PBS and Fe_3_O_4_@HA nanoparticles at the Fe concentrations of 0.025, 0.05, 0.1, 0.2, and 0.4 mM, respectively. The morphologies of the nanoparticle groups were similar to those treated with PBS, and no obvious changes were observed. **(B)** *In vitro* cytotoxicity of Fe_3_O_4_@HA nanoparticles measured by MTT assay (*n* = 3 per group). Nanoparticles show no cytotoxicity until Fe concentration is at 0.2 mM. A slight decrease (less than 10%) in cell viability compared with the PBS group was observed at a Fe concentration of 0.4 mM. **(C)** Investigation of targeting ability and quantitative determination of cellular uptake of Fe_3_O_4_@HA nanoparticles using Hela cells (*n* = 3 per group). The HA blocked group is a negative control, in which HeLa cells were pretreated with HA to saturate the CD44 receptor and then incubated with Fe_3_O_4_@HA nanoparticles. The normal Fe_3_O_4_@HA group showed higher cellular uptake than the HA-blocked group at all concentrations, possibly through an active targeting mechanism. Data are presented as mean ± SD.

In order to function as imaging agents for living cells, the nanoparticles have to get into cells and also be compatible with cells. We therefore evaluated the cytotoxicity and cellular uptake of Fe_3_O_4_@HA nanoparticles. The nanoparticles at different Fe concentrations (0.025–0.4 mM) were incubated with HeLa cells for 24 h at 37°C. The morphologies of HeLa cells treated with Fe_3_O_4_@HA were observed by phase contrast microscopy. The images (Figure 7A) showed that the morphologies of the nanoparticle groups were similar to those treated with PBS, and no obvious changes were observed, indicating that Fe_3_O_4_@HA nanoparticles are compatible with cells under the current concentration range. This observation was further verified by *in vitro* cytotoxicity studies. The viabilities of HeLa cells were measured after incubation with Fe_3_O_4_@HA nanoparticles (0.025, 0.5, 0.1, 0.2, and 0.4 mM) for 24 h by MTT assay. Clearly, the Fe_3_O_4_@HA nanoparticles did not exert any cytotoxicity until the Fe concentration was at 0.2 mM (Figure 7B). Further increase in the Fe concentration to 0.4 mM slightly decreased the cell viability by less than 10% compared with the cells treated with PBS. These results, together with the observation of cell morphology, demonstrated that Fe_3_O_4_@HA nanoparticles are biocompatible and almost non-cytotoxic at the Fe concentration up to 0.4 mM.

Since HA has high binding affinity with the CD44 receptor that is overexpressed in HeLa cells, we next assessed the targeting ability of HA toward the model HeLa cell line. Cells were treated with PBS and Fe_3_O_4_@HA nanoparticles at concentrations of 0.025, 0.5, 0.1, 0.2, and 0.4 mM. After 4 h of incubation, the cells were washed with PBS three times, digested, centrifuged, and resuspended in PBS for cell counting. Cells were further lysed, and the Fe uptake was quantified by inductively coupled plasma-atomic emission spectroscopy (ICP-AES). The negative control group was created by pretreating HeLa cells with HA (10 μM in DMEM) for 2 h to saturate the CD44 receptor and then incubating with Fe_3_O_4_@HA nanoparticles. As shown in Figure 7C, the HeLa cells showed a dose-dependent cellular uptake of Fe_3_O_4_@HA. The amount of nanoparticles entered the cells increased with increasing Fe concentration in both the Fe_3_O_4_@HA group and the negative control HA-blocked group. More importantly, the normal Fe_3_O_4_@HA group showed higher cellular uptake compared with the HA-blocked group at all concentrations. Quantitively, the amounts of Fe uptake in the Fe_3_O_4_@HA group were 1.7, 2.6, 1.5, 1.6, and 1.9 folds compared with those in the HA-blocked group at the incubation concentrations of 0.025, 0.5, 0.1, 0.2, and 0.4 mM, respectively (Figure 7C). These observations suggested that the HA corona on the surface of Fe_3_O_4_@HA nanoparticles can mediate the specific interactions with the CD44 receptor expressed by HeLa cells and realize an active targeting mechanism. The results also perfectly elaborate on the design principle of the work.

### *In Vitro* MRI of HeLa Cells

**FIGURE 8:**
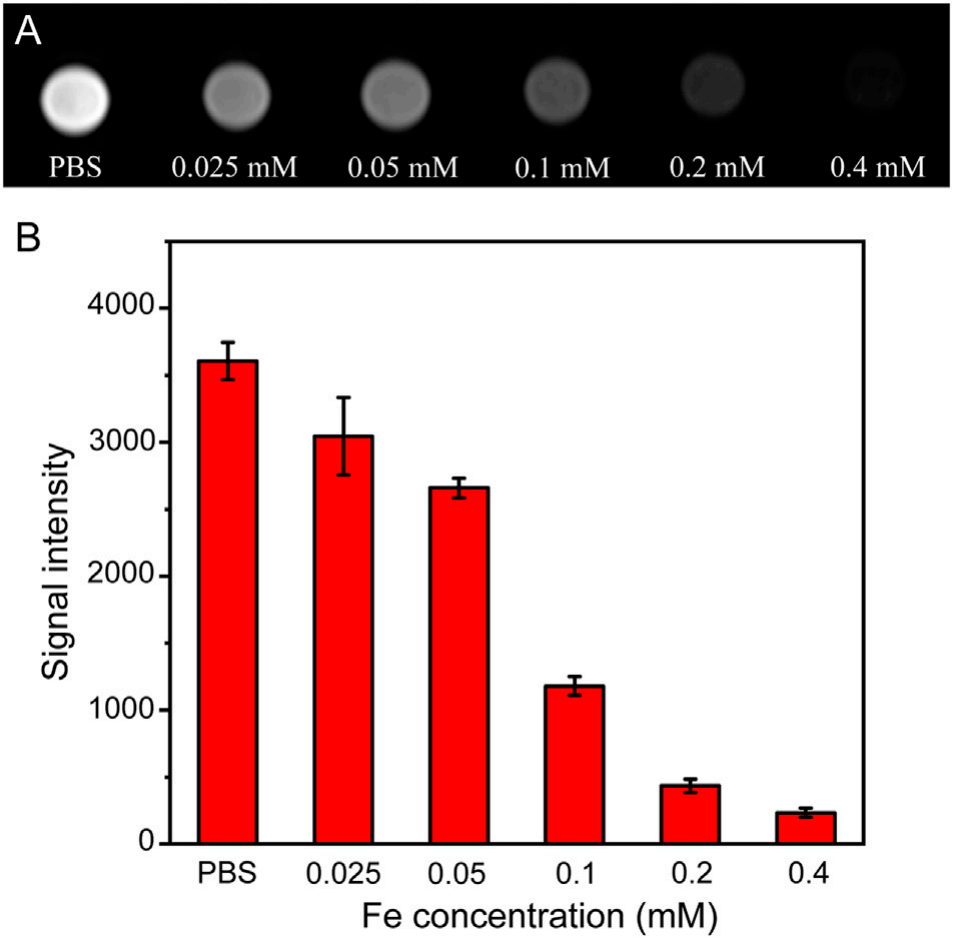
**(A)** *T*_2_-weighted *in vitro* MRI of HeLa cells treated with PBS and Fe_3_O_4_@HA nanoparticles at concentrations of 0.025, 0.05, 0.1, 0.2, and 0.4 mM for 6 h (*n* = 3). **(B)** Corresponding signal intensity of MRI results. Data are calculated from three parallel measurements and presented as mean ± SD.

To verify the feasibility of using Fe_3_O_4_@HA nanoparticles as contrast agents, we next performed an *in vitro* MRI study. HeLa cells were co-incubated with Fe_3_O_4_@HA nanoparticles at various Fe concentrations (0.025, 0.5, 0.1, 0.2, and 0.4 mM) for 6 h at 37°C with PBS as a control. Furthermore, *T*_2_-weighted MRI of the HeLa cells was performed and analyzed. As shown in Figure 8A, the MR images of the cells treated with Fe_3_O_4_@HA presented lower signal intensity than the PBS control group. The signal also exhibited a dose-dependent behavior, which decreased with the increase in the Fe concentration (Figure 8B). These results indicated the effective role of Fe_3_O_4_@HA nanoparticles in providing adequate imaging contrast, and the dose-dependent decrease in signal intensity is probably a result of the concentration-dependent cellular uptake.

### *In Vivo* MRI in a Xenografted Tumor Model in Mice

**FIGURE 9:**
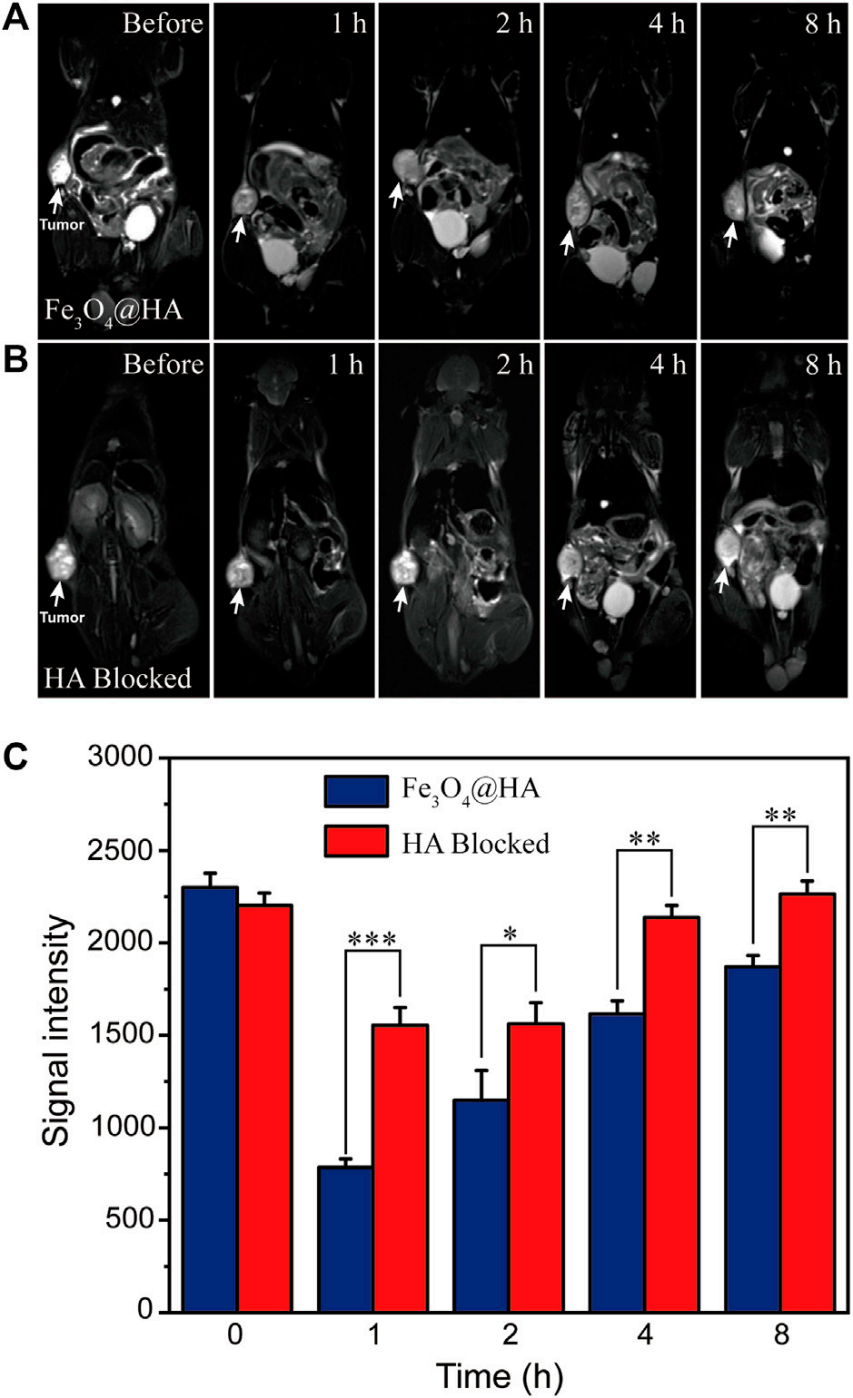
**(A,B)** *T*_2_-weighted *in vivo* MRI of nude mice (*n* = 3) bearing HeLa tumor before and after administration of Fe_3_O_4_@HA nanoparticles. The HA-blocked group **(B)** is a negative control, in which HA was intratumorally injected to saturate the CD44 receptor 1 h before administration of Fe_3_O_4_@HA nanoparticles. **(C)** Comparison of the corresponding signal intensity of MRI results of Fe_3_O_4_@HA and HA-blocked groups (*n* = 3 per group). The significant differences in the intensity indicate that Fe_3_O_4_@HA nanoparticles achieved more effective tumor targeting in the HA active state than in the HA blocked state. Data are presented as mean ± SD and analyzed by one-way ANOVA (0.01 < *p ≤ 0.05; **p ≤ 0.01; ***p ≤ 0.001).

After demonstrating the possibility of *in vitro* MRI in cells, we further investigated the use of Fe_3_O_4_@HA nanoparticles for *in vivo* MRI in a HeLa xenografted tumor model. When the tumor reached a size of around 150 mm^3^, Fe_3_O_4_@HA nanoparticles were administered intravenously at Fe concentration of 10 mM in 100 μL PBS. MRI was then performed at 1, 2, 4 and 8 h post-injection. In the group of blocked HA, it (100 μM in 50 μL PBS) was intratumorally injected to saturate the CD44 receptor 1 h before administration of nanoparticles. The *T*_2_-weighted MR images of Fe_3_O_4_@HA and HA-blocked groups are shown in Figures 9A,B, respectively. We found that the MR signals in both groups decreased after injection. Based on more quantitative assessment (Figure 9C), we found that the highest contrast enhancement happened at 1 h post injection and then gradually recovered. In the Fe_3_O_4_@HA group, the signals decreased by 66, 50, 30, and 20% at 1, 2, 4, and 8 h post-injection, respectively, compared with that before injection. However, in the HA-blocked group, the signals only decreased by 29% at 1 h post injection and had already recovered back to similar intensity as that before injection at 4 h. Direct comparison between Fe_3_O_4_@HA and HA-blocked groups showed significant differences, indicating that Fe_3_O_4_@HA nanoparticles achieved more effective tumor targeting in the HA active state than in the HA-blocked state. The slight decrease in the MR intensity in the HA-blocked group could be a result of passive targeting because of the EPR effect, and the recovery of the intensity could be mainly attributed to the metabolism process that cleared nanoparticles out of the body, but the significantly lower MR signals in the Fe_3_O_4_@HA group than in the HA blocked group at the same time point clearly indicated the role of the HA-mediated specific tumor targeting pathway in addition to the EPR effect. These results are consistent with our *in vitro* cellular uptake studies, which again confirmed that Fe_3_O_4_@HA nanoparticles could target the tumor through an active targeting mechanism, realizing more effective MRI both *in vitro* and *in vivo*.

## Conclusion

In this article, we created the HA-stabilized Fe_3_O_4_ nanoparticles simply through electrostatic interactions. The adhesion of HA onto the surface of bare Fe_3_O_4_ nanoparticles contributes to the dispersibility and stability of colloidal aggregates. The formed Fe_3_O_4_@HA nanoparticles are compatible with cancer cells and RBC suspension in the studied concentration range. The relatively high *r*_2_ relaxivity is a result of the thin-layered HA corona that allows better penetration of water molecules into the particle core. Our findings showed that Fe_3_O_4_@HA nanoparticles are able to enter into HeLa cells overexpressing the CD44 receptor through a specific cell targeting pathway in the HA active state compared with the HA-blocked state. Furthermore, the created Fe_3_O_4_@HA nanoparticles can be used as effective nanoprobes for targeted MRI of both HeLa cells *in vitro* and xenografted HeLa tumors *in vivo*. We hope that our findings will eventually contribute to building up the general design principle of the targeted delivery of nanoparticles for both cancer diagnosis and therapy.

## Materials and Methods

### Materials

Hyaluronic acid (6 KDa) was purchased from Zhenjiang Dong Yuan Biotechnology Corporation (Zhenjiang, China). Iron (III) chloride hexahydrate (FeCl_3_.6H_2_O), iron (II) chloride tetrahydrate (FeCl_2_.4H_2_O), and sodium hydroxide were purchased from Aladdin Ltd. (Shanghai, China). 3-(4,5Dimethylthiazol-2-yl)-2,5-diphenyltetrazolium bromide (MTT) was supplied by Thermo Fisher Scientific, Ltd. (Waltham, MA). Fetal bovine serum (FBS), Dulbecco’s modified Eagle’s medium (DMEM), penicillin-streptomycin, and trypsin were obtained from Gibco Life Technologies Co. (Grand Island, NY). Regenerated cellulose dialysis membranes with a molecular weight cut-off (MWCO) of 50 KDa were acquired from Fisher (Pittsburgh, PA).

### Synthesis of Fe3O4@HA Nanoparticles

The nanoparticles were synthesized *via* a facile controlled co-precipitation method. Briefly, 30 mg of HA was first dissolved in 25 ml Milli-Q water (resistivity higher than 18.2 MΩ cm, a Milli-Q Plus 185 water purification system (Millipore, Bedford, MA)) and stirred in a three-neck round-bottom flask to obtain a homogeneous solution. The solution was degassed with bubbling N_2_ to remove O_2_ and heated to 80°C. Then, 0.36 g of FeCl_3_.6H_2_O and 0.132 g of FeCl_2_.4H_2_O were dissolved in 7.5 ml O_2_-free Milli-Q water and added to the HA solution. After stirring for 15 min under N_2_ protection, 10 ml of O_2_-free Milli-Q water containing 1 g of NaOH was quickly added to the mixture under mechanical stirring of 1200 rpm. The reaction was continued for another 2 h at 80°C. After cooling down to room temperature, the black solution was 1) centrifuged at a low speed of 600 rpm to get rid of the big aggregations and 2) magnetically separated and re-dispersed in the Milli-Q water. The obtained solution was further dialyzed (molecular weight cut-off at 50 KDa) against Milli-Q water for 2 days to remove free ions and HA. A small quantity of the Fe_3_O_4_@HA solution was subjected to freeze-drying, and the leftover was stored at 4°C.

### Characterization of Fe_3_O_4_@HA Nanoparticles

The crystalline structure of the Fe_3_O_4_ and Fe_3_O_4_@HA nanoparticles was characterized by X-ray diffraction (XRD) in a 2θ range of 20–80°, using a D/max 2550 PC X-ray diffractometer (Japan, Rigaku Cop.) with Cu Kα radiation (*λ* = 0.154,056 nm) at 40 kV and 200 mA. Fourier transform infrared (FTIR) spectra of Fe_3_O_4_ and Fe_3_O_4_@HA nanoparticles were obtained by using a Nexus 670 spectrometer (Thermo Nicolet Corporation, Madison, WI). Thermogravimetric analysis (TGA) was performed in a temperature range of 30–900°C with a heating rate of 20°C/min under nitrogen using a TG209 F1 (NETZSCH Instruments Co., Ltd., Germany) thermogravimetric analyzer.

### Size and Stability Measurements

The TEM samples were prepared by adding 10 μL of Fe_3_O_4_@HA nanoparticle (Fe concentration 0.1 mM) solution onto a carbon-film-coated copper grid (400 square mesh, Electron Microscopy Sciences, Hatfield, PA, United States), and the excess solution was wicked with a filter paper. The grid was air-dried before imaging and was then imaged using a FEI Tecnai 12 TWIN transmission electron microscope (100 kV). A SIS Megaview III wide-angle CCD camera was used to acquire the TEM images. The samples for dynamic light scattering measurements were prepared at a Fe concentration of 0.2 mM, and then, the hydrodynamic size of Fe_3_O_4_@HA nanoparticles was measured by a Malvern Zetasizer Nano ZS model ZEN3600 (Worcestershire, U.K.) equipped with a standard 633-nm laser. Three repeated measurements for each sample were determined to give the average values and standard deviations.

### Determination of *T*_2_ Relaxivity

The Fe concentration of Fe_3_O_4_@HA nanoparticles was previously determined using the Leeman Prodigy Inductively Coupled Plasma-Atomic Emission Spectroscopy (ICP-AES) system (Hudson, NH03051). Sample solutions at Fe concentrations of 0.00156, 0.00313, 0.00625, 0.0125, 0.025, 0.05, and 0.1 mM were prepared by dilution of stock solution before measurements. *T*_*2*_ relaxometry was performed by using a 0.5-T NMI20-Analyst NMR Analyzing and Imaging system (Shanghai Niumag Corporation, China). Instrumental parameters are as follows: a point resolution of 156 mm × 156 mm, section thickness of 0.6 mm, TR of 4000 ms, TE of 60 ms, and number of excitation of 1. The *T*_*2*_ relaxivity was determined by linear fitting of 1/*T*_*2*_ *vs.* Fe concentration.

### Hemolysis Assay

Mouse blood (1.5 ml) collected from the inner canthus vein plexus was mixed with 3.5 ml of PBS. Pure red blood cells (RBCs) were obtained *via* repeated centrifugation/redispersion processes (2000 rpm, 10 min, three times). The RBCs were then diluted with 5 ml of PBS for further use. A measure of 100 μL of the obtained RBC suspension was mixed with 900 μL PBS (negative control), water (positive control), and Fe_3_O_4_@HA nanoparticle solution in PBS at various Fe concentrations (0.025, 0.05, 0.1, 0.2, 0.4, 0.8, and 1.6 mM). After 2 h incubation at 37°C, sample solutions were centrifuged at 10,000 rpm for 15 min. The absorbance of the supernatant for each sample at 540 nm was then measured *via* a Lambda 25 UV-Vis spectrophotometer (PerkinElmer, Boston, MA). The hemolysis rate was calculated as follows: hemolysisrate(%)=(A(sample, 540 nm)-A(negative, 540 nm))/(A-(positive, 540 nm)A(negative, 540 nm))×100% .

### Cell Culture, Morphology, and *In Vitro* Cytotoxicity

HeLa cells were purchased from the Institute of Biochemistry and Cell Biology (the Chinese Academy of Sciences, Shanghai, China) and cultured in DMEM containing 5% FBS and 1% antibiotics at 37°C and 5% CO_2_.

HeLa cells (10,000 cells/well) were seeded in 96-well plates overnight. The Fe_3_O_4_@HA nanoparticles at various Fe concentrations (0.025, 0.05, 0.1, 0.2, and 0.4 mM) were next incubated with the HeLa cells for another 24 h in 200 μL of DMEM. HeLa cells treated with PBS were used as a control. The morphology of HeLa cells was further observed by phase contrast microscopy (Leica DM IL LED inverted phase contrast microscope) at a magnification of 200 times.

*In vitro* cytotoxicity was further quantitively confirmed by the MTT assay. Similar to the protocols described earlier, HeLa cells (10,000 cells/well) were seeded in 96-well plates overnight. The Fe_3_O_4_@HA nanoparticles at various Fe concentrations (0.025, 0.05, 0.1, 0.2, and 0.4 mM) were then incubated with the HeLa cells for another 24 h in 200 μL DMEM. Furthermore, the cells were rinsed three times with PBS and then incubated in 100 μL of FBS-free DMEM medium containing 10% MTT for 4 h. After removal of the medium, the MTT assay was performed according to the manufacturer’s instructions. For each concentration, three parallel wells were measured to give the average values and standard deviations.

### Cellular Uptake of Fe3O4@HA in HA Active and Blocked States

HeLa cells (2 × 10^6^ cells/well) were seeded in 6-well plates for overnight adherence. The seeded plates were divided into two groups, and the cell medium was replaced on the second day. One group was replaced with fresh DMEM medium, and the other group was replaced with fresh DMEM containing HA (10 μM) that pre-saturates the overexpressed CD44 receptor in HeLa cells. After incubation for another 2 h, the medium was removed, and the cells were washed with PBS three times. Then, the cells were further incubated with fresh DMEM containing Fe_3_O_4_@HA nanoparticles at various Fe concentrations (0.025, 0.05, 0.1, 0.2, and 0.4 mM) for 4 h at 37°C. Next, the cells were washed three times with PBS, digested by trypsinization, centrifuged (1000 rpm, 5 min), and resuspended in PBS for cell counting. The remaining cells were centrifuged (1000 rpm, 5 min) and lysed using 0.5 ml aqua regia solution (nitric acid/hydrochloric acid, v/v = 1:3) for one day. Finally, we diluted the samples with PBS, and the cellular uptake of the Fe_3_O_4_@HA nanoparticles was evaluated by inductively coupled plasma-atomic emission spectroscopy (ICP-AES).

### *In Vitro* MRI of Cancer Cells

The animal study protocol was approved by the Ethics Committee of Scientific Research and Clinical Trials of the First Affiliated Hospital of Zhengzhou University. HeLa cells (5 × 10^6^ cells/flask) were seeded into 25 cm^2^ culture flasks with 5 ml DMEM overnight at 37°C. On the second day, Fe_3_O_4_@HA nanoparticles at different Fe concentrations (0.025, 0.05, 0.1, 0.2, and 0.4 mM) in 5 ml fresh medium were replaced. After 6 h incubation, the HeLa cells were rinsed three times with PBS, digested by trypsinization, centrifuged (1000 rpm, 5 min), and resuspended in 1 ml (containing 0.5% agarose) PBS in a 2-ml eppendorf tube. The *T*_*2*_-weighted MRI of HeLa cells was performed on a 3.0 T Signa HDxt superconductor clinical MR system (GE Medical Systems, Fairfield, CT). 2D spin-echo MR images were obtained with the parameters of 2 mm slice thickness, TR/TE 2000/96.2 ms, FOV 6 × 6 cm, and 256 × 256 matrix.

### *In Vivo* MRI of the Tumor Model

For the experiment, 6-week-old female BALB/c nude mice were purchased from SPF (Beijing) Biotechnology Co., Ltd. HeLa cells (2 × 10^6^ in 100 ml PBS) were subcutaneously injected into the left back of the mice. After 3–4 weeks, when the tumor reached a size ∼150 mm^3^, the mice were randomly divided into two groups (*n* = 3 per group). One group was intratumorally injected with HA (100 μM) in 50 μL PBS 1 h before the injection of Fe_3_O_4_@HA contrast agents. Then, both groups of mice were anesthetized by an intraperitoneal injection of pentobarbital sodium (40 mg/kg). The Fe_3_O_4_@HA nanoparticles (Fe concentration = 10 mM, in 100 μL PBS) were then intravenously injected into mice. The *in vivo* tumor MRI studies were conducted at different time points (1, 2, 4, and 8 h post injection) using a 3.0 T Signa HDxt superconducting clinical MR system attached with a custom-built animal receiver coil. 2D spin-echo MR images were obtained with the parameters of 2 mm slice thickness, TR/TE 2000/96.2 ms, FOV 6 × 6 cm, and 256 × 256 matrix. The *T*_*2*_-weighted MR images before administration were also obtained as controls.

## Data Availability

The raw data supporting the conclusion of this article will be made available by the authors without undue reservation.
